# Teacher Perspectives on Standing Desks in the Classroom: A Community-Engaged Approach to Reducing Sedentary Behavior in Youth

**DOI:** 10.7759/cureus.106145

**Published:** 2026-03-30

**Authors:** Katie L Wasserstein, Caitlin M Byerly, Eric W Schaefer, Mark E Benden, Deepa L Sekhar

**Affiliations:** 1 Department of Pediatrics, Penn State College of Medicine, Hershey, USA; 2 Department of Public Health Sciences, Penn State College of Medicine, Hershey, USA; 3 Department of Environmental and Occupational Health, Texas A&M University School of Public Health, College Station, USA

**Keywords:** activity permissive learning environment, community-engaged research, school-based interventions, standing desk, teacher opinion

## Abstract

Objective

Reducing sedentary behavior in youth is a global health priority, with classroom-based interventions, such as standing desks, offering potential benefits. This study used community-engaged research (CEnR) methods to inform the development of a standing desk intervention by assessing teacher perspectives on implementation barriers and relevant study outcomes in K-12 schools.

Methods

An electronic survey was administered on February 7-8, 2024, to Pennsylvania teachers via the Penn State PRO Wellness Healthy Champions program, which provides online wellness resources to schools, along with the publication of a monthly newsletter. Eligible respondents taught K-12 students in brick-and-mortar schools. The 48-item survey assessed demographics, use of activity-permissive learning environments (APLE) including standing desks, willingness to trial APLE, and acceptability of study outcomes.

Results

Of 81 initial respondents, 48 met the inclusion criteria. Teachers were predominantly white (44/48, 91.7%), female (34/48, 70.8%), and > 40 years old (26/48, 54.2%). Most (45/48, 93.8%) used traditional desks; only (14/48, 29.2%) currently use APLE. A majority (30/48, 62.5%) expressed willingness to trial standing desks. Barriers included cost, space limitations, and lack of administrative support. Teachers prioritized improvements in student concentration (22/48, 45.8%) over health-related outcomes. Questionnaire-based outcome measures were most acceptable, while biometric measures (e.g., BMI, blood pressure) and finger-prick blood collection were viewed less favorably. Logistic regression found no demographic predictors of willingness to trial standing desks.

Conclusion

Teachers' interest in classroom standing desks centers on potential benefits for student focus and behavior, with hesitancy around health data collection and logistical constraints. These findings guided the research team's CEnR approach to school partnerships, resulting in the inclusion of concentration-related outcomes and planning for barriers in the study design. Engaging educators early in the research process supported alignment between research goals and community priorities, enhancing the feasibility of a school-based standing desk intervention.

## Introduction

In 2020, the World Health Organization released guidelines on the reduction of sedentary behavior in children and adolescents, recognizing the detrimental effects of sedentary behavior on youth fitness, cardiometabolic health, adiposity, behavior, and sleep [1]. Activity-permissive learning environments (APLE) may be one way to reduce sedentary behavior. APLE refers to classroom design that gives students the ability to move while being engaged in classroom learning, compared to the traditional classroom with forward-facing chairs and desks in a row-and-column layout [2]. APLE has gained popularity in classrooms both to provide children who struggle to sit still the freedom to move without disrupting the class and to make the classroom less "institutional" [3,4]. APLE includes, but is not limited to, standing desks, pedal desks, wobble stools, and bean bag chairs [5]. Studies demonstrate a positive relationship between the use of APLE seating, specifically standing desks, and a reduction in sedentary behavior [6].

This survey formed the initial stakeholder-engagement step of a broader community-engaged research (CEnR) study. CEnR methods involve collaboration between researchers, the community (i.e., schools), and key partners to consider study design and outcomes of interest to all parties, leading to the greatest application of research results [7]. While researchers were interested in the impact of standing desks on childhood body mass index (BMI) and weight trajectory, consistent with the principles of CEnR, it was understood that engaging key partners (teachers) to understand their concerns would be instrumental to project success. The primary objective of this study was to assess K-12 teachers' willingness to trial standing desks and identify perceived barriers to implementing APLE. Secondary objectives were to characterize current APLE use and evaluate teachers' preferences for specific behavioral, academic, and biometric outcome measures to inform the design of a subsequent school-based intervention trial.

## Materials and methods

Survey development

This study involved an electronically administered teacher survey regarding APLE with a focus on standing desks. Survey questions were designed collaboratively by the research team and then pilot tested with five educators. Researchers adjusted the question/response language based on the feedback received. For example, the graphics used on the survey were updated based on this feedback for better clarity.

The final teacher survey comprised ­­48 questions. The survey began with screening questions to confirm eligibility: 1) a teacher 18 years or older (any gender), 2) actively teaching at least one grade from kindergarten to 12th grade, and 3) in a brick-and-mortar (non-remote) school building.

Following confirmation of eligibility, teacher and school demographic information were requested without identifying information. Teachers were asked about perceptions and use of APLE, with a graphic to reference various APLE options. The graphic was published with a prior manuscript [5]. Teachers were asked to provide input on their willingness to participate (without commitment) in a study of standing desks and what the "selling point" of participation might be for them. Finally, teachers were queried on the acceptability of potential study outcomes. The Institutional Review Board approved this study on January 26, 2024 (STUDY00023940).

Data collection

The survey link was open from February 7-8, 2024, with responses collected through Research Electronic Data Capture (REDCap), a secure database for conducting research studies [8]. Survey completion constituted participant consent to participate. Participants were compensated with a $10 gift card. Based on available funds, the goal was to obtain a maximum of 50 completed surveys. The link itself was distributed to Pennsylvania teachers via our team's existing Healthy Champions program electronic newsletter, which is sent out monthly. The Healthy Champions program interfaces with approximately 500 school districts across the Commonwealth [9]. The February newsletter was sent to 5,616 recipients, and 1,693 recipients opened the message. Recipients are not only teachers, as other school professionals (i.e., school nurses) engage with the Healthy Champions program. The survey was advertised midway through the newsletter as “Flexible Seating Survey” with an image of a wobble stool and read as, “Are you a classroom teacher? Share your opinions about using activity-permissive learning environments (APLE, i.e., standing desks) in this brief survey. Participants will receive a $10 electronic Amazon gift card.” No reminders were sent, as the survey was only open for two days.

Data analysis

Descriptive data were summarized. For grades taught, elementary school was defined as kindergarten through fifth grade, middle school as sixth to eighth grade, and high school as ninth to 12th grade. Four teachers (8.3%) who taught multiple levels (e.g., middle and high school) were classified according to the youngest age they taught. For the number of students in the classroom, the midpoint was used (rounded up) for six teachers (12.5%) who reported a range of students (i.e., if a teacher reported 20-25 students, then 23 was used). Teacher willingness to trial standing desks was based on the survey question, “Would you be interested in trialing the use of stand-up desks?” In the logistic regression, answers of “no” and “maybe” were combined and compared to “yes” to clearly understand teacher and school demographics associated with definite interest in standing desks. Separate logistic regression models were fit for five variables: current APLE use, teacher age, grades taught, classroom size, and school size. For these models, we used complete case analysis, which was reasonable since only one response was missing for teacher age and for classroom size. A multivariable model was not considered because the sample size and number of events were too small to feasibly fit the model. We used R (version 4.5.0; R Development Core Team, Vienna, Austria) for the statistical analysis [10].

## Results

Demographics

Of the 81 interested teachers who began the survey, 48 met the inclusion criteria to participate and completed the survey. Teachers were primarily white (44/48, 91.7%), non-Hispanic (43/48, 89.6%), female (34/48, 70.8%), over 40 years old (26/48, 54.2%), and with a master's degree or above (39/48, 81.3%). Of the teachers surveyed, the majority (38/48, 79.2%) taught in middle and high school buildings. Most teachers (37/48, 77.1%) taught class sizes of 20 students or fewer. The average school size was 578.8 students (SD: 219.9).

Use of APLE

Table 1 details teachers' opinions on APLE in the classroom. Most teachers (45/48, 93.8%) currently use traditional classroom desks. A minority (14/48, 29.2%) currently use APLE, and most (29/48, 60.4%) have never done so. Teachers with previous experience with APLE but not currently using it (3/48, 6.3%) identified barriers, including cost (2/3, 66.7%), lack of administrator support (2/3, 66.7%), and lack of classroom space (1/3, 33.3%).

**Table 1:** Pennsylvania teacher opinion on Activity Permissive Learning Environments (APLE) in the classroom

Traditional and APLE seating used in the classroom (check all that apply; N=48). Participants were provided with a graphic for this question that was published in a prior manuscript [5]	N (%)
Traditional Desk	45 (93.8)
Balance Ball	2 (4.2)
Standing Desk	4 (8.3)
Wobble Stool	6 (12.5)
Bean Bag Chair	8 (16.7)
Pedal Desk	0 (0)
Other	13 (27.1)
Decline to Answer	0 (0)
Which of the following best describes your classroom? (N=48)	
We currently use APLE	14 (29.2)
We have used APLE in the past, but are not currently using it	3 (6.3)
We have never used APLE	29 (60.4)
Decline to answer	2 (4.2)
You indicated that you have never used APLE in your classroom. Why not? (select all that apply; N=29)	
Cost of flexible seating equipment is a barrier	22 (75.9)
Lack of administrator support/approval	6 (20.7)
I feel it negatively impacts classroom behavior	3 (10.3)
My students don’t like it	1 (3.4)
Classrooms is not big enough for the equipment	9 (31.0)
Other	4 (13.8)
Decline to answer	1 (3.4)
Would you be interested in trialing the use of standing desks in your current classroom? (N=48)	
Yes	30 (62.5)
No	9 (18.8)
Unsure	9 (18.8)
Based on your perspective as a teacher, what would be the most appealing benefit to be gained from standing desks? Phrased another way, what would be the most valuable selling point for teachers? (N=48)	
Improved concentration	22 (45.8)
Improved behavior	9 (18.8)
Improved classroom management	4 (8.3)
Improved academic performance	8 (16.7)
Improved heart health or fitness	5 (10.4)

Most teachers (30/48, 62.5%) were willing to trial standing desks in their classrooms. Eighteen respondents who were unsure or unwilling to trial standing desks wrote free-response answers for their perceived barriers to participation. Four teachers cited a lack of student buy-in, three cited classroom size, three cited student behavioral issues, two cited administrative and time issues, two cited student physical inability to use the desks, and four did not cite a specific reason. Representative comments included the following: “(students) would not be receptive to the change,” “not all students would be able to handle the responsibility of a standing desk,” “time required for teachers to participate,” and “students… cannot stand for periods of time due to disabilities or injuries.”

Table 2 summarizes teacher opinions on potential outcomes for a standing desk study. Teachers felt that the greatest benefit of standing desk use would be in improved student concentration (22/48, 45.8%). Teachers were most comfortable with the use of questionnaire-based outcomes, such as a sleep questionnaire (34/48, 70.8%), a concentration questionnaire (37/48, 77.1%), and a mental wellness/mood questionnaire (37/48, 77.1%). Teachers also felt that data on academic performance and behavior (30/48, 62.5%) would be well-received. Objective measures, such as BMI (27/48, 56.3%) and blood pressure (26/48, 54.2%), were anticipated to be less well-received. Blood collection via finger prick had the lowest ratings.

**Table 2:** Pennsylvania teacher input on the potential outcomes for a standing desk study (N=48) ^a^Via finger prick

Potential study outcomes	Responses N (%)			
	Yes	Unsure	No	Decline to Answer
Body mass index	27 (56.3)	10 (20.8)	9 (18.8)	2 (4.2)
Waist Circumference	20 (41.7)	14 (29.2)	12 (25.0)	2 (4.2)
Blood Pressure	26 (54.2)	13 (27.1)	7 (14.6)	2 (4.2)
Accelerometer Data (watch worn to track activity level)	17 (35.4)	24 (50.0)	6 (12.5)	1 (2.1)
Cholesterol^a^	6 (12.5)	23 (47.9)	17 (35.4)	2 (4.2)
Hemoglobin A1C (measure of blood sugar in the past 3 months)^a^	6 (12.5)	23 (47.9)	17 (35.4)	2 (4.2)
Sleep Questionnaire	34 (70.8)	10 (20.8)	3 (6.3)	1 (2.1)
Concentration Questionnaire	37 (77.1)	10 (20.8)	0 (0)	1 (2.1)
Mental Wellness/Mood Questionnaire	37 (77.1)	9 (18.8)	1 (2.1)	1 (2.1)
Academic Performance and Behavior	30 (62.5)	14 (29.2)	1 (2.1)	3 (6.3)

Logistic regression

Logistic regression models found that no teacher or school demographics (current APLE use, teacher age, grades taught, classroom size, and school size) were significantly associated with willingness to use standing desks (Table 3).

**Table 3:** Logistic regression model for variables influencing teacher willingness to use standing desks in the classroom

Variable	OR (95% CI)	p-value
Current APLE use		
Yes	2.05 (0.62, 6.76)	0.24
No (ref)		
Teacher Age		
≥40 years	0.80 (0.24, 2.66)	0.72
<40 years (ref)		
Grades taught		
High school	0.18 (0.03, 1.10)	0.06
Middle school	0.70 (0.11, 4.48)	0.71
Elementary school (ref)		
Classroom Size (continuous by 1 student increase)	0.91 (0.73, 1.14)	0.43
School Size (continuous by 100 student increase)	0.93 (0.71, 1.22)	0.6

## Discussion

The results of this study indicate that, while most teachers continue to utilize traditional classroom seating, a majority are willing to trial APLE, specifically standing desks. Teachers are most interested in the benefits of standing desks on student concentration. Barriers include cost, lack of administrator support, and lack of classroom space. Paired with previously published data from a parent and child survey, these data successfully informed the research team's CEnR approach with Pennsylvania school districts for partnership on a classroom standing desk study [5].

The overall positive response from parents, teachers, and children regarding trialing standing desks reinforced the research team's selection of this type of APLE for further research [5]. Teacher opinions on APLE demonstrate greater concordance compared to other current educational topics, with greater variability in teacher opinions, e.g., classroom cell phone use, and the teaching of potentially controversial subjects [11,12]. The survey study results directly informed how the research team approached school districts for study partnership.

First, understanding teachers' primary interest in the benefits of standing desks on concentration prompted a discussion of this outcome with the school district administration. This led to the inclusion of time-on-task assessments as a proxy measure of concentration in the study proposal [13]. Second, the research team was also interested in health-related outcomes about which teachers expressed more hesitancy (e.g., BMI). However, BMI measurements are typically managed by the school nurse (versus teachers) [14]. Thus, during discussions with school district administration, the team was thoughtful to discuss the rationale behind the request for BMI outcomes and request feedback from the school nurse about the logistics of obtaining BMI measurements. Finally, the teacher-identified barriers, including desk costs and installation, were discussed with the school administration and included in the research budget. Concerns regarding classroom and desk size were approached by physically going to potential partnering school districts and measuring current desks against possible standing desk models, with teacher feedback on space and classroom layout. Thus, teacher input informed priority outcomes and anticipated barriers, but feasibility must be empirically evaluated during the intervention trial.

Limitations of this study include a small sample size of Pennsylvania teachers, which may limit the generalizability of the findings to a broader perspective, such as teachers nationwide. The study was underpowered to detect demographic predictors; nonsignificant results should not be interpreted as evidence of no association. The survey was based on a convenience sample of Pennsylvania teachers who received the Healthy Champions newsletter and who chose to respond to the survey. Those who receive and respond to a survey advertisement in a wellness newsletter may be more willing than those who do not to try standing desks. Additionally, respondents were from a demographically homogenous sample: predominantly female, greater than 40 years old, and non-Hispanic white. While this is consistent with the demographics of Pennsylvania teachers and relevant to the schools approached for study partnership, it may not be representative [15]. The survey was open for only two days, as the maximum number of responses was achieved. A short survey window could prohibit a wider variety of responses. Survey development was also limited in that no cognitive testing or psychometric analyses were completed.

Finally, it is acknowledged that this survey was an initial step to ground the proposed research in key partner perspectives as the start of a more extensive, richer plan for CEnR.

## Conclusions

Consistent with the principles of CEnR, engaging educators from the start of the research process allowed for early alignment of research goals and community priorities, enhancing the feasibility of the planned school-based standing desk intervention. This study provides exploratory, descriptive data on teacher perceptions that may not generalize to all populations but may help guide the design of a future school-based intervention. The results of this survey of Pennsylvania teachers informed the start of engagement efforts with school districts and the development of a more detailed, thoughtful engagement plan as part of the resulting study proposal. Should funding be successfully obtained, we look forward to continuing to leverage CEnR principles in the successful completion and dissemination of this research work.

## Appendices

Appendix 1

You are being invited to volunteer to participate in a research study. This summary explains information about this research.

Teachers, of any gender, who are over the age of 18 years and teach at a physical (brick and mortar) school are eligible to complete this survey. The survey will take approximately 5 minutes to complete. Participants will be compensated with a $10 electronic gift card to the provided email address after completing the survey. There is a risk of loss of confidentiality if your information or your identity is obtained by someone other than the investigators, but precautions will be taken to prevent this from happening. The confidentiality of your electronic data created by you or by the researchers will be maintained as required by applicable law and to the degree permitted by the technology used. Absolute confidentiality cannot be guaranteed. Your confidentiality will be maintained to a degree permitted by the technology used for the online survey. Specifically, no guarantees can be made regarding the interception of data sent via the internet by any third parties. You will receive $10 for your participation in this research study. If you elect not to complete the survey, you will not be compensated. The payment will be provided by an electronic gift card that will be distributed through the participant's electronic mail address, which will be obtained through a separate REDCap survey.

If you have questions, complaints, or concerns about the research, you should contact Dr. Deepa Sekhar at XXX-XXX-XXXX. If you have questions regarding your rights as a research subject or concerns regarding your privacy, you may contact the Human Research Protection Program at XXX-XXX-XXXX. Your participation is voluntary, and you may decide to stop at any time. You do not have to answer any questions that you do not want to answer.

Your participation implies your voluntary consent to participate in the research.

Table 4 presents the Activity Permissive Learning Environments (APLE) Teacher Survey.

**Table 4:** Activity Permissive Learning Environments (APLE) Teacher Survey There is a figure referenced throughout the survey. As stated in the manuscript body, the graphic was previously published in a companion parent-child standing desk survey manuscript [5].

Eligibility Screening	
Question	Response Options
Are you a classroom teacher?	1, Yes
	2, No
What grade(s) do you teach? (select all that apply)	1, Kindergarten
	2, 1st
	3, 2nd
	4, 3rd
	5, 4th
	6, 5th
	7, 6th
	8, 7th
	9, 8th
	10, 9th
	11, 10th
	12, 11th
	13, 12th
	99, Decline to answer
What subject(s) do you teach?	(open text)
Do you teach in-person at a "brick and mortar" school (i.e., in person)?	1, Yes
	2, No
Survey Questions	
Question	Response Options
1. Which of the traditional and flexible seating options shown above do you use in your classroom (check all that apply)*?	1, A (traditional desk)
	2, B (balance ball)
	3, C (stand-up desk)
	4, D (wobble desk)
	5, E (bean bag chair)
	6, F (pedal desk)
	7, Other
	99, Decline to answer
1b. (If ‘7’ in Q1) What other non-traditional seating options have you used in your class?	(open text)
2. Which of the following best describes your classroom?	1, We currently use APLE
	2, We have used APLE in the past, but are not currently using it
	0, We have never used APLE
	99, Decline to answer
2b. (If ‘2’ in Q2) You indicated that you previously used APLE in your classroom, but are not currently using it. Why not? (select all that apply)	1, Cost of flexible seating equipment is barrier
	2, Lack of administrator support/approval
	3, I feel it negatively impacts classroom behavior
	4, My students don't like it
	5, Classroom is not big enough for the equipment
	88, Other
	99, Decline to answer
2b1. (If ‘88’ in Q2b) Please describe the other reason(s) you no longer utilize APLE.	(open text)
2c. (If ‘0’ in Q2) You indicated that you have never used APLE in your classroom. Why not? (select all that apply)	1, Cost of flexible seating equipment is barrier
	2, Lack of administrator support/approval
	3, I feel it negatively impacts classroom behavior
	4, My students don't like it
	5, Classroom is not big enough for the equipment
	88, Other
	99, Decline to answer
2c1. (If ‘88’ in Q2c) Please describe the other reason(s) you have never utilized APLE.	(open text)
School Demographics	
3. Please check all grades served by your school building:	1, Kindergarten
	2, 1^st^
	3, 2^nd^
	4, 3^rd^
	5, 4^th^
	6, 5^th^
	7, 6^th^
	8, 7^th^
	9, 8^th^
	10, 9^th^
	11, 10^th^
	12, 11^th^
	13, 12^th^
4. Approximately how many students are in your school building?	(open text)
5. Approximately how many students are in each classroom?	(open text)
6. Do students in your school currently have daily planned time for physical activity (recess equivalent not including PE)?	1, Yes
	0, No
	99, Decline to answer
7. How much and often do students have physical education at your school? Report as minutes/cycle (i.e., 90 minutes per 6 day cycle)	(open text)
Proposed APLE Study	
Previous research studies examined the use of standing desks with adults and found improvements in cardiometabolic risk factors (body mass index, cholesterol, blood sugar). In 2015 Vallecito Elementary in California replaced all of its standard desks with flexible seating, specifically stand-up desks, with great success. However, the use of flexible seating, including standing desks, has not been extensively studied in children.	
We hope to better understand this topic through a future research study. A bit about the proposed study... We would conduct our study by randomly assigning students in each participating school to either standing desks or traditional desks by classroom or team depending on school structure/scheduling. Participating schools would have the cost of the stand-up desks covered, and retain them when the study concluded. We would request some data points regularly collected about students, such as weight and height for routine body mass index screening. The collection of any additional student health data points would be supported by the study team, in concert with a school nurse. We hope to understand if using standing desks impacts a variety of health factors in participating students. To do this, we would need to gather additional data from students, such as those items listed below. To collect this type of data we would require a student's parent to consent. Please select the items below that you think would be feasible to collect in a school setting (assuming parent consent was obtained).	
8. Body Mass Index (BMI) (This would include weight and height measurements that are already collected by the school annually)	1, Yes, seems feasible
	2, Unsure
	0, No, would not advise
	99, Decline to answer
9. Waist Circumference (Measuring tape around child's waist. Would measure this with BMI screening as it is more accurate for some kids than BMI)	1, Yes, seems feasible
	2, Unsure
	0, No, would not advise
	99, Decline to answer
10. Blood Pressure (Would measure at the time of BMI screening)	1, Yes, seems feasible
	2, Unsure
	0, No, would not advise
	99, Decline to answer
11. Accelerometer Data (Kids would wear a watch (similar to a Fitbit) for 1 week that tracks their heart rate, steps, and sleep. We would get a daily step count and heart rate variability)	1, Yes, seems feasible
	2, Unsure
	0, No, would not advise
	99, Decline to answer
12. Cholesterol (This would be done onsite with a finger prick (similar to a diabetic checking blood sugar) NOT a blood draw)	1, Yes, seems feasible
	2, Unsure
	0, No, would not advise
	99, Decline to answer
13. Hemoglobin A1C (A measure of blood sugar in the past 3 months, this would be done onsite with a finger prick)	1, Yes, seems feasible
	2, Unsure
	0, No, would not advise
	99, Decline to answer
14. Sleep Questionnaire (Would be emailed to students through the school and ask about hours of sleep and trouble falling asleep)	1, Yes, seems feasible
	2, Unsure
	0, No, would not advise
	99, Decline to answer
15. Concentration Questionnaire (Similar to above, but would ask about ability to focus in the classroom setting)	1, Yes, seems feasible
	2, Unsure
	0, No, would not advise
	99, Decline to answer
16. Mental Wellness/Mood Questionnaire (Similar to above, but would query your child's mood and emotions)	1, Yes, seems feasible
	2, Unsure
	0, No, would not advise
	99, Decline to answer
17. Academic Performance and Behavior (Based on both teacher report and an outside expert monitoring the class)	1, Yes, seems feasible
	2, Unsure
	0, No, would not advise
	99, Decline to answer
18. Are there any additional health items you think we should measure?	(open text)
19. We'd be interested in making the students part of the project by involving them in thinking like real scientists. This could include co-designing a series of lessons with school science/STEM teachers. (For example, What are the student's thoughts of what will happen in the study? How would they design the study? What might be weaknesses with the study itself?) Do you think teachers would be open to having students "be the scientists" by co-designing a lesson plan to involve them in thinking through study design and outcomes? No identifiable student data would be shared.	1, Yes
	2, Unsure
	0, No
	99, Decline to answer
20. With the background information shared, if this opportunity were presented to your school, would you be interested in trialing the use of stand-up desks in your current classroom? Note: this question is not seeking commitment for participation, only your level of interest in participation in this type of study.	1, Yes
	2, Unsure
	0, No
	99, Decline to answer
20b. (If ‘2’ or ‘0’ in Q20) Please describe your perceived barriers to participation.	(open text)
21. Based on your perspective as a teacher, what would be the most appealing benefit to be gained from standing desks? Phrased another way, what would be the most valuable selling point for teachers?	1, Improved concentration
	2, Improved behavior
	3, Improved classroom management
	4, Improved academic performance
	5, Improved heart health or fitness
22. Do you think parents would be open to having their students be part of such a study?	1, Yes
	2, Unsure
	0, No
	99, Decline to answer
22b. (If ‘2’ or ‘0’ in Q22) Please describe your anticipated barriers to parent support.	(open text)
23. Please share any additional considerations regarding the feasibility of implementing the described research in your classroom.	(open text)
Demographic Data	
What is your current age?	1, < 20
	2, 20-29
	3, 30-39
	4, 40-49
	5, 50 or older
	99, Decline to answer
What is your race?	1, American Indian or Alaskan Native
	2, Asian
	3, Black or African American
	4, Native Hawaiian or Pacific Islander
	5, White
	88, Some Other Race
	89, Two or More Races
	99, Decline to answer
What is your ethnicity?	1, Hispanic or Latino
	2, Not Hispanic or Latino
	99, Decline to answer
What is your gender?	1, Male
	2, Female
	3, Non-binary
	99, Decline to answer
What is your highest level of education attained?	1, Did not complete high school
	2, High school
	3, Some college
	4, College degree
	5, Master's degree or higher
	99, Decline to answer
